# Headache education in Africa: a narrative review of educational programs, clinical practice implications, and policy opportunities

**DOI:** 10.3389/fneur.2026.1862942

**Published:** 2026-07-08

**Authors:** Nicholas Aderinto, Thomas Oyediran, Emmanuela Ojoagefu Egwu, Emmanuel Kodizuru Chukwuemeka, Meron Yitna Teshome, Oluwafisayo Tosin Olasupo, Faith Adedayo Adejumo

**Affiliations:** 1 Department of Medicine, Ladoke Akintola University of Technology, Ogbomoso, Nigeria; 2 College of Health, Ladoke Akintola University of Technology, Ogbomoso, Nigeria; 3 Department of Medicine and Surgery, Bowen University, Iwo, Osun, Nigeria; 4 College of Medicine, University of Ibadan, Ibadan, Nigeria; 5 School of Public Health, Washington University, St. Louis, AR, United States; 6 Ryazan State Medical University, Ryazan, Russia

**Keywords:** Africa, headache disorders, headache education, neurology training, primary care

## Abstract

Headache disorders rank among the most prevalent and disabling neurological conditions globally, yet they remain severely under-recognized and undertreated across Africa. Educational deficits at every level of the health system perpetuate misdiagnosis, delayed care, and preventable disability. We conducted a narrative review by searching PubMed, Scopus, Web of Science, Embase, and AJOL. Eligible sources included peer-reviewed articles, systematic reviews, policy documents, and gray literature relating to headache burden, education, and care delivery in Africa. Thematic synthesis was applied across four domains: epidemiology and burden; current educational status; impact of education on clinical practice; and policy, multidisciplinary, and culturally responsive approaches. Africa bears a substantial but under-quantified headache burden driven by data gaps, cultural stigma, limited specialist access, and inadequate training of frontline providers. Structured educational interventions, including the Education in Headache to Healthcare Providers in Africa (EHHPA) symposium, the IHS Regional Outreach Program in Malawi, and the Global Migraine Aid initiative in Kenya, have reported improvements in self-assessed diagnostic confidence and knowledge scores among participants, though robust patient-level outcome data remain limited. Task-shifting to non-physician clinicians and community health workers is feasible and effective. Culturally responsive approaches, engagement of traditional healers, and gender-sensitive frameworks are essential for equitable reach. Education is the lever for improving headache care across Africa. Sustained investment in workforce training, community awareness, digital platforms, policy integration, and locally generated research is urgently needed. Aligning headache education with the WHO Intersectoral Global Action Plan (IGAP) and national non-communicable disease strategies offers the most scalable pathway to reducing the headache burden across the continent.

## 1 Introduction

Headache disorders are among the most common and disabling neurological conditions worldwide (1, 2). Global Burden of Disease (GBD) estimates indicate that nearly 3 billion people are affected, with headache disorders accounting for approximately 4.6% of total years lived with disability (YLDs) (1). Migraine is the leading contributor to disability, despite tension-type headache (TTH) being more prevalent; migraine is, in fact, the primary cause of disability in individuals under 50 years of age (2, 3). Importantly, the burden of headache disorders is not confined to high-income settings: prevalence and disability appear broadly similar across low-, middle-, and high-income regions (2, 4).

Despite these global insights, sub-Saharan Africa (SSA) remains one of the least epidemiologically characterized regions (5, 6). Available evidence nevertheless indicates a substantial and under-recognized burden. A systematic review estimated migraine prevalence at approximately 5.6%, representing nearly 56 million individuals across the continent (7). Higher prevalence is observed among women, urban populations, and younger adults, disproportionately affecting individuals in their most economically productive years (6, 7). Migraine ranks among the leading causes of YLDs in Africa and contributes significantly to neurological disability (1, 8). However, robust national data are lacking; most evidence derives from small, heterogeneous studies, limiting generalizability across Africa's diverse settings (5, 6). Secondary headaches, including those related to infectious diseases and medication-overuse headache (MOH), are likely more prevalent in this context yet remain poorly quantified (9).

The broader socioeconomic impact is considerable. Africa's population is young and rapidly expanding, with a growing proportion entering the workforce (10). Headache disorders in this demographic are associated with reduced productivity, absenteeism, and impaired quality of life (11). Yet headaches are frequently normalized, stigmatized, or attributed to non-medical causes, leading many individuals to self-medicate or seek traditional care rather than formal health services (12). This contributes to delayed diagnosis and persistent, avoidable disability.

Limitations in the healthcare system further exacerbate these challenges. SSA has an estimated neurologist density of approximately 0.3 per million population, with some countries lacking specialist services entirely (13, 14). As a result, most patients with headache are managed in primary or community care settings by non-specialist providers, including general practitioners, nurses, pharmacists, and community health workers, many of whom have received limited training in headache medicine (14). This contributes to under-recognition, misdiagnosis, and reliance on symptomatic rather than evidence-based management (4, 15).

Resource constraints compound these gaps. Health systems in many African countries prioritize communicable diseases and maternal–child health, leaving headache disorders relatively neglected. Access to essential medicines is often limited, and migraine-specific therapies remain inconsistently available or unaffordable (4, 14). Diagnostic resources and referral pathways are scarce, further restricting effective care.

In this context, education is a critical, scalable intervention. Strengthening training for healthcare professionals across all levels, alongside improving patient and community awareness, has the potential to enhance recognition, diagnosis, and management of headache disorders in Africa (4, 14). Initiatives such as the Education in Headache to Healthcare Providers in Africa (EHHPA) program demonstrate the feasibility of structured, context-specific educational strategies to build capacity and improve care delivery (16, 17).

This review, therefore, aims to highlight how strengthening education can improve clinical practice, inform policy, and ultimately enhance patient outcomes across Africa. We also propose a comprehensive set of evidence-based recommendations for policymakers, educators, and international partners.

## 2 Methods

This narrative review synthesizes current knowledge on headache education, clinical practice, and policy implications in Africa. A literature search was conducted across PubMed, Scopus, Web of Science, Embase, and AJOL using combinations of the following terms: “headache disorders,” “migraine,” “Africa,” “healthcare education,” “neurology training,” “primary care,” and “health systems.” Additional sources were identified through manual reference list searches and organizational reports from the World Health Organization, EHHPA, and the International Headache Society (IHS).

Studies were eligible for inclusion if they addressed headache burden, education, or care delivery in LMICs, particularly in Africa. Eligible source types included peer-reviewed articles, systematic reviews, policy reports, and gray literature published in English. Gray literature (organizational reports, program websites, and webinars) was included where no peer-reviewed equivalent was available and is explicitly distinguished from peer-reviewed evidence throughout the manuscript. Studies were excluded if they were not available in English, did not pertain to Africa or another LMIC context, or addressed headache solely as a secondary symptom without educational relevance. Two authors (NA and EOE) independently selected sources for inclusion; disagreements were resolved through discussion and consensus among the authorship group. No strict date restrictions were applied; however, priority was given to recent and high-impact evidence. Studies were selected based on relevance to the review's four thematic domains: (1) current epidemiological burden and educational status; (2) impact of education on clinical practice; (3) policy implications; and (4) opportunities for culturally responsive and multidisciplinary approaches. Findings were narratively synthesized within these domains.

## 3 Results

The literature search identified sources addressing headache education in Africa. Following screening for relevance, 10 sources were included in the thematic synthesis. These comprised seven peer-reviewed studies reporting primary data and three gray literature sources describing educational programs and implementation initiatives. Findings were synthesized across *four* thematic domains: (1) current status of headache education in Africa, (2) impact of education on clinical practice, (3) policy implications, and (4) opportunities for multidisciplinary and culturally sensitive approaches. Sources were excluded if they were duplicates, not published in English, or did not address headache education in Africa.

### 3.1 Current status of headache education in Africa

Despite the high prevalence of headache disorders across Africa, headache education remains inadequate at all levels of the health system.

#### 3.1.1 Training and knowledge gaps among healthcare professionals

Most headache patients across Africa are not seen by neurologists or specialists, but by general practitioners, clinical officers, and nurses who have received little or no formal training in headache medicine. A WHO assessment across 47 African countries found only 1.55 healthcare professionals per 1,000 people, well below the WHO threshold of 4.45 per 1,000, with only four countries meeting this benchmark (18). Even where diagnostic knowledge exists, management gaps persist. A study in Burkina Faso found that while 80% of general practitioners demonstrated adequate knowledge of ICHD-3 migraine criteria, over half still referred headache patients to specialists primarily for access to better treatment options (19). Headache medicine is largely absent from undergraduate medical curricula, and a WHO global survey identified poor training in headache care for healthcare workers as one of the core barriers to care in LMICs (4).

The deficit in specialist training is alarming. A survey of neurology trainees across 19 SSA countries revealed persistent gaps in resources, mentorship, and structured curricula, with implications for the pipeline of future headache specialists (20). The consequence is a workforce at every level that is insufficiently equipped to recognize, diagnose, or manage the full spectrum of headache disorders encountered in clinical practice.

#### 3.1.2 Public awareness and stigma

Low public awareness further deepens the clinical gap. In SSA, headache disorders are frequently dismissed as “normal” or perceived as minor because they are episodic, nonfatal, and noncontagious, a perception that results in stigmatization and prevents many individuals from seeking care despite significant pain and disability (12). Cultural beliefs exacerbate this problem: in many rural African communities, headaches are attributed to supernatural causes such as evil spirits or curses, and traditional healers are consulted in preference to medical professionals (12, 21). In Mali, 68.8% of patients with neurological disorders, including headache, consulted a traditional medicine practitioner before accessing biomedical care, with this proportion rising to 85.5% among illiterate patients (21).

Structural stigma also operates within health systems. Patients who miss work or social obligations because of migraine are often labeled “lazy,” and those with normal neuroimaging are told their pain is psychological, findings highlighted by the Migraine Outreach webinar series (2025) (22). These dynamics discourage help-seeking, delay diagnosis, and entrench a cycle of inadequate care (12).

#### 3.1.3 Review of existing educational programs

Several initiatives have begun to address these gaps (Table 1). The most established is the Education in Headache to Healthcare Professionals in Africa (EHHPA), a free annual virtual symposium co-organized by the Global Patient Advocacy Coalition for Headache (GPACH), the African Association of Neurological Sciences (AFAN), and the World Federation of Neurology (WFN). Its growth has been remarkable: the 2022 edition attracted 839 registrants from 77 countries, representing a 51% increase from the prior year (16). By 2024, participation had grown to 1,318 from 102 countries, with 79% of participants reporting increased confidence in distinguishing migraine from other headache disorders and 75% reporting improved diagnostic confidence (17). These figures reflect participant self-report and should be interpreted as indicative of perceived learning gain rather than confirmed clinical impact.

**Table 1:** Key educational programs addressing headache care in Africa and LMICs.

Source	Setting	Main finding	Type of education intervention	Methodology for education and training	Ref.
EHHPA 2023	Pan-African (virtual)	Reached 1,318 participants from 102 countries in 2024; most reported improved confidence in migraine diagnosis and headache assessment.	Remote	Standalone course (annual symposium)	(16)
IHS-ROPE–DREAM Partnership	Malawi	Structured training improved headache knowledge among non-physician clinicians; competency thresholds rose from 24 to 56%.	In-person teaching and training	Two or more lessons (iterative program, 2022 and 2025)	(24)
Global migraine aid initiative	Kenya	Demonstrated feasibility of training non-physician clinicians in migraine diagnosis and treatment within primary care.	In-person teaching and training with task shifting	Not reported	(23)
Mercy migraine management program	United States (LMIC-relevant model)	Educational intervention associated with substantial reductions in headache frequency and improved quality of life.	Not reported	Not reported	(25)
Migraine outreach webinar series	Rwanda, Nigeria, Zambia, United States (virtual)	Identified task-shifting and primary care training as key strategies for expanding headache care capacity.	Remote	One lesson (single webinar)	(22)
EHHPA 2024	Pan-African (virtual)	Provider education improved diagnostic accuracy, increased treatment initiation, and reduced unnecessary investigations.	Remote	Standalone course (annual symposium)	(17)
Dabilgou et al.	Burkina Faso	Most general practitioners recognized migraine diagnostic criteria, but treatment-related barriers remained common.	Not reported	Not reported	(19)
Mateen et al.	19 SSA countries	Highlighted deficiencies in neurology training, mentorship, and specialist workforce development.	Not reported	Not reported	(20)
Maiga et al.	Mali	Traditional healers were frequently consulted before biomedical services, emphasizing the importance of culturally responsive education.	Not reported	Not reported	(21)
Dodd-Glover et al.	Sub-Saharan Africa	Documented major system-level barriers, including limited specialist services and poor access to migraine-specific therapies.	Not reported	Not reported	(14)

AMD, association of migraine disorders; AFAN, African association of neurological sciences; DREAM, disease relief through excellent and advanced means; EHHPA, education in headache to healthcare providers in Africa; GPACH, global patient advocacy coalition for headache; IHS, international headache society; NPC, non-physician clinician; ROPE, regional outreach program; WFN, world federation of neurology.

In Kenya, the Global Migraine Aid initiative, led by Prof. Rami Burstein, has focused on training non-physician providers to diagnose and treat migraine using sumatriptan and other locally available medications (23). In 2025, Migraine Outreach and the Association of Migraine Disorders convened a webinar featuring panelists from Rwanda, Nigeria, Zambia, and the United States, reaching consensus that headache education for primary care workers represents “the lowest hanging fruit” for improving patient outcomes, and that task-shifting managing up to 80% of headache care at the primary level is essential given the critical neurologist shortage (22).

Another significant initiative is the IHS Regional Outreach Program (ROPE), which, in 2022 and 2025, partnered with the Disease Relief through Excellent and Advanced Means (DREAM) program to deliver structured, in-person headache training to non-physician clinicians in Blantyre, Malawi (24). These programs collectively demonstrate both the feasibility and scalability of structured headache education in low-resource African settings.

#### 3.1.4 Barriers to headache education

Headache education in Africa is impeded by several interrelated obstacles. At the systemic level, headache disorders are not recognized as medical conditions in many LMIC health systems, and advocacy capacity is limited by the scarcity of locally trained experts (14, 26). Reduced headache education results from a lack of advocacy groups, restricted access to care, and limited availability of prescription drugs (12). Preventive treatments are rarely used, triptans remain exceptional rarities, and care is largely confined to simple analgesics, a situation that directly correlates with high rates of medication-overuse headache (4). Cultural beliefs, gender inequities, language barriers, and low health literacy further attenuate the reach of educational programs, particularly in rural and low-connectivity settings (12, 21).

### 3.2 Impact of education on clinical practice

Africa's population of working-age adults faces a disproportionately high headache burden, with migraine and other primary headache disorders affecting an estimated 10%−15% of the general population and contributing substantially to regional disability (9). Given the dire shortage of specialists, approximately 0.3 neurologists per million in SSA (13, 14), most headache care is necessarily delivered in primary care settings by general practitioners and non-physician clinicians (NPCs) who often lack formal training in headache medicine (27). Education serves as the essential vehicle for bridging this gap, transforming clinical practice and informing policy to improve patient outcomes even in resource-constrained settings (28).

#### 3.2.1 Diagnostic accuracy

Provider education directly addresses the pervasive problem of misdiagnosis. In many SSA contexts, headaches are managed as accompanying symptoms of prevalent infectious diseases such as malaria or HIV, with primary headache disorders frequently overlooked (29). Evidence demonstrates that structured training significantly improves diagnostic precision. In Estonia, a controlled interventional study showed that after a brief educational program, general practitioners shifted from non-specific diagnostic labels toward ICD-10–compliant, disease-specific diagnoses (15). Concurrently, GP-requested investigations (laboratory tests and radiographs) decreased from 26 to 4%, and GP-initiated treatment rose from 58 to 81%, with a marked increase in the use of evidence-based treatment options (15).

The IHS ROPE in Malawi similarly reported that NPCs achieved significant knowledge gains in recognizing diagnostic criteria for primary headaches and identifying “red flags” for secondary disorders (24). By the second program iteration, the proportion of participants achieving a sufficient knowledge threshold rose from 24 to 56% (24). Such findings confirm that education enables clinicians to apply specific, accurate diagnoses, reducing missed and mismanaged cases across clinical practice.

#### 3.2.2 Treatment optimization

Education fundamentally alters how clinicians manage headache disorders. Trained providers are better equipped to implement the IHS “Essential” treatment framework, designed specifically for resource-limited settings where access to advanced medications or neuroimaging is restricted (30, 31). Focusing on cost-effective strategies such as early use of simple analgesics (aspirin or ibuprofen), trained clinicians can achieve meaningful population-level health gains at low cost (32). Education also equips providers to identify and manage MOH, a growing concern in Africa given the unregulated availability of analgesics (6, 33).

#### 3.2.3 Patient outcomes

Clinical improvements translate directly into patient wellbeing. The Mercy Migraine Management Program (MMMP) demonstrated that a low-cost educational intervention for both providers and patients resulted in 46% of participants achieving a 50% or greater reduction in headache frequency over 12 months (25). Participants also reported significant improvements in quality of life and headache-related disability (25, 34). Furthermore, educational programs improve patient satisfaction by equipping providers to better answer questions and set realistic management goals (35).

In Africa, the impact of these outcomes is profound: better management reduces lost-productivity burden, which is estimated to cost some economies up to 2% of their GDP (36). The EHHPA program, reaching clinicians across 102 countries, and with 79% reporting increased diagnostic confidence, provides meaningful evidence that task-shifting headache care to NPCs is an effective, replicable strategy for expanding access to neurological care across the continent (17).

### 3.3 Policy implications

Education is a critical lever for bridging the gap between clinical knowledge and the systemic transformation required to strengthen health sectors (37). When healthcare professionals, patients, and communities are better informed, the standard of clinical decision-making rises, creating downstream pressure for institutional and policy change (38).

One critical mechanism by which education influences policy is through the adoption and implementation of clinical guidelines (39). Evidence-based guidelines provide structured frameworks for care (30, 31). However, without training frontline healthcare workers, these guidelines remain chronically underutilized (40). Educational initiatives directed at primary care providers who first encounter headache patients can enhance understanding of these frameworks and promote standardized, evidence-based care a priority that is especially acute in low-resource settings where simplified, tiered approaches can guide efficient resource utilization (41).

Educating healthcare professionals fosters empowerment and drives advocacy for improved headache care systems (38). When providers are equipped to recognize and articulate the burden and impact of headache disorders, they become advocates for access to essential medications, for inclusion of headache in NCDs strategies, and for allocation of funding to neurological services (42). Patient education similarly reduces misconceptions, encourages health-seeking behavior, and creates visible demand for services that policymakers must address (43).

Despite the high prevalence and disability associated with headache disorders in Africa, few countries have specific policies addressing them (44). Educational programs can generate awareness and data to inform policy development and build workforce capacity. Professional societies, including national neurological associations and global bodies such as the IHS, can drive change through collaboration to develop standardized curricula, training programs, and certification pathways (31, 41). These collaborative initiatives also facilitate the sharing of best practices and the adaptation of global standards to local African contexts.

Aligning headache education with the WHO Intersectoral Global Action Plan on Epilepsy and Other Neurological Disorders (IGAP) strengthens policy relevance (45, 46). When headache education is integrated into IGAP-aligned national strategies, headache care is embedded within broader brain health initiatives, improving coordination, increasing funding opportunities, and strengthening political commitment (46). The WHO also emphasizes that headache disorders should be taught alongside other neurological conditions (47), allowing healthcare workers to build broader competencies while developing specific headache management skills embedded across undergraduate training, postgraduate education, and continuing medical education (CME) programs.

### 3.4 Opportunities for multidisciplinary and culturally sensitive approaches

About 56 million people in Africa live with migraine, creating a major social and economic burden, yet the condition remains underrecognized and underfunded in health systems (3, 7). Limited training, low awareness, cultural beliefs, and language and literacy barriers further weaken care pathways. With an estimated 270 million people in countries with fewer than five neurologists (48), access to care is critically constrained. Addressing these gaps requires culturally grounded approaches that reflect the social determinants shaping headache experiences across African communities.

#### 3.4.1 Integrating cultural practices and traditional medicine

Across SSA, cultural beliefs and traditional healing systems strongly influence care-seeking behavior. In Mali, 68.8% of patients with neurological disorders, including headache, consulted traditional medicine practitioners before biomedical care, rising to 85.5% among illiterate patients (21). These patterns reflect rational navigation of trusted, accessible, and culturally resonant systems, not ignorance of biomedicine. Educational programs that dismiss traditional medicine risk alienating communities; more effective approaches integrate cultural perspectives while clearly communicating evidence-based headache diagnosis and treatment (49).

Evidence from South Africa shows that traditional practitioners hold deep community trust and can serve as bridges to biomedical care when engaged respectfully (50). Curricula co-developed with clinicians, traditional healers, and community leaders are more likely to produce durable behavioral change (49). This integrative approach aligns with global frameworks: the WHO IGAP and the Africa Health Workforce Agenda both call for holistic, multidisciplinary strategies (45, 51). The IHS-ROPE-DREAM partnership in Malawi exemplifies how culturally relevant training can strengthen primary care capacity in SSA (24).

#### 3.4.2 Community health workers and patient advocacy

Community health workers (CHWs) remain underused as resources for headache education and care delivery. Africa CDC notes that CHWs anchor community health systems and reach remote areas as frontline responders (52). Adding headache-specific training to CHW competency frameworks could substantially expand the delivery of evidence-based care (52, 53). Ethiopia's Health Extension Program demonstrates that structured community engagement can address complex disease management needs in low-resource rural settings (53).

Patient engagement and advocacy are equally important. Advocacy by individuals with migraine during peak working years can shift policy priorities and increase funding for headache care. GPACH highlights the EHHPA 2024 symposium as a strong model for patient-centered coalition-building, with similar coalitions emerging across Africa (54). Multidisciplinary teams integrating physicians, nurses, pharmacists, psychologists, and community advocates consistently improve chronic disease outcomes and offer a proven framework for headache management in African health systems (27).

#### 3.4.3 Gender, language, and literacy considerations

Gender, language, and literacy remain major barriers to equitable headache education. Women experience higher migraine prevalence and YLD rates globally, including in Africa (55, 56), yet face greater structural barriers to care. Sacco and Porreca have called for sex-inclusive research designs and gender-sensitive training programs to bridge the gap in headache medicine (57). Language and literacy barriers further limit the reach of educational materials; programs must invest in local-language adaptation and non-text-based formats to reach populations with low health literacy (58).

Intersectionality must guide the design of headache education, as emphasized by the American Headache Society's Equity, Diversity, and Inclusion (EDI) Advisory Council (56). Models such as EHHPA, IHS-ROPE, and WHO Africa's competency-based curricula demonstrate real momentum; what is now needed is scale, sustained funding, and political commitment to make culturally sensitive, multidisciplinary headache education the standard across Africa (59–64).

## 4 Recommendations and future directions

Despite the high burden of headache disorders, care remains inadequate across Africa. Educational gaps represent a fundamental and often overlooked determinant of this inadequacy. A set of strategic, multi-level recommendations is proposed below (Table 2) to strengthen headache education, improve clinical practice, inform policy development, and enhance patient outcomes.

**Table 2:** Strategic recommendations for strengthening headache education and care in Africa.

Domain	Recommendation	Responsible actor(s)	Timeline
Health system	Integrate headache disorders into national NCDs strategies and mental/brain health plans aligned with WHO IGAP	Ministries of Health	Short–medium term
Education and training	Embed headache medicine in undergraduate and postgraduate medical, nursing, and pharmacy curricula	Universities/Medical Councils	Medium term
Workforce development	Scale task-shifting models: train NPCs, community health workers, and pharmacists in headache recognition and first-line management	Health ministries, NGOs	Short term (urgent)
Medicines access	Ensure triptans and evidence-based preventive agents are included on national essential medicines lists and are affordable	Regulatory agencies, WHO	Medium term
Research and surveillance	Establish national headache registries; mandate systematic evaluation of all educational interventions with patient-level outcomes	Research institutions, funders	Ongoing
Community engagement	Co-develop culturally responsive public education campaigns with community leaders, traditional healers, and patient advocates	NGOs, professional societies	Short term
Digital innovation	Expand virtual CME platforms (e.g., EHHPA model) and leverage mobile health tools for training in low-connectivity settings	Tech partners, WFN, IHS	Short term
International collaboration	Institutionalize North–South and South–South partnerships for curriculum co-development, clinical mentorship, and funding	IHS, WFN, African neurology societies	Medium term

CME, continuing medical education; EHHPA, education in headache to healthcare providers in Africa; IGAP, intersectoral global action plan; IHS, international headache society; NCDs, non-communicable diseases; NGO, non-governmental organization; NPC, non-physician clinician; WHO, World Health Organization; WFN, world federation of neurology.

Building on these priorities, primary care providers should be retrained to recognize headache disorders and escalate or treat as appropriate. The EHHPA program, organized free of charge and reaching over 1,300 participants from 102 countries in 2024 (17), illustrates both the demand and the feasibility of structured, cost-free training at scale; more such programs are urgently needed. Beyond healthcare workers, low community-level awareness remains a major challenge. Many patients do not recognize headache disorders as medical conditions, leading to self-medication, delayed presentation, and poor adherence (12, 21). Structured public education through grassroots campaigns, radio, television, and school-based programs is needed to improve early presentation and outcomes.

Integration within national health policies, rather than isolated initiatives, is essential for sustained improvements in headache care. Aligning efforts with the WHO IGAP on epilepsy and other neurological disorders can support national strategies and promote consistency with international best practices (45, 46). A major barrier to advancing headache care in Africa is the scarcity of high-quality locally generated data. Future efforts should prioritize establishing headache registries, systematically evaluating training programs, investigating diagnostic delays, and measuring patient-level outcomes. Given resource constraints, strong partnerships between local institutions, international organizations, and professional bodies are essential, and digital learning platforms and social media offer cost-effective ways to extend reach while maintaining quality.

## 5 Limitations

This review has limitations that should be considered when interpreting its findings. Country-level epidemiological data across Africa are scarce and heterogeneous, meaning that many estimates of headache burden rest on a small number of studies from a limited set of countries; findings may not be generalizable across the continent's diverse health systems, languages, and cultural contexts. Also, several educational programs described in this review are represented only by gray literature such as organizational reports and program webpages, which have not undergone independent peer review; the outcomes they report (participant numbers, self-reported confidence gains) reflect program monitoring rather than rigorously evaluated clinical evidence. Outcome data are predominantly participant self-reports of knowledge and confidence; objective measures of diagnostic accuracy and, crucially, patient-level clinical outcomes remain largely absent from the literature, limiting conclusions about the downstream impact of educational interventions on patient health.

## 6 Conclusion

Headache disorders represent a significant yet under-recognized source of disability across Africa, driven by gaps in education, awareness, and health system capacity. This review demonstrates that insufficient healthcare provider training and limited community knowledge lead to misdiagnosis, ineffective management, and preventable suffering. Educational interventions have been shown to enhance diagnostic accuracy, optimize treatment, and improve patient outcomes even in low-resource environments. Education is also a driver of policy change and health system strengthening, empowering providers and patients to generate demand, evidence, and advocacy that can unlock broader systemic reforms. Multidisciplinary approaches that engage traditional healers, community health workers, patient advocates, and gender-responsive frameworks are necessary to achieve equitable impact at scale. Increasing headache education across Africa is not merely a clinical imperative; it is a viable, scalable, and cost-effective strategy for reducing neurological disability and improving population health across the continent. With sustained investment, political commitment, and international collaboration, it is achievable.
